# Unveiling a New 2D Semiconductor: Biphenylene-Based InN

**DOI:** 10.1021/acsomega.4c03511

**Published:** 2024-06-24

**Authors:** José A. S. Laranjeira, Nicolas Martins, Pablo A. Denis, Julio Sambrano

**Affiliations:** † Modeling and Molecular Simulation Group, School of Sciences, São Paulo State University (UNESP), Bauru 17033-360, Brazil; ‡ Computational Nanotechnology, DETEMA, Facultad de Química, UDELAR, CC 1157, 11800 Montevideo, Uruguay

## Abstract

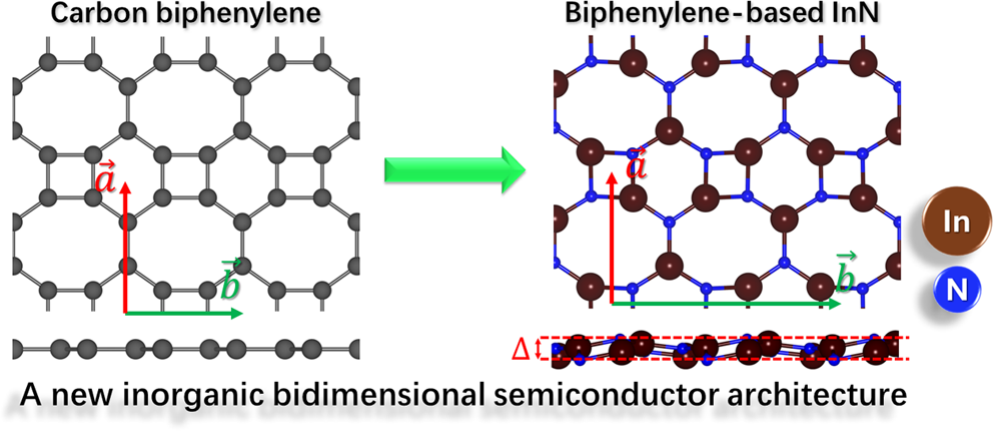

The two-dimensional (2D) materials class earned a boost in 2021 with biphenylene synthesis, which is structurally formed by the fusion of four-, six-, and eight-membered carbon rings, usually named 4-6-8-biphenylene network (BPN). This research proposes a detailed study of electronic, structural, dynamic, and mechanical properties to demonstrate the potential of the novel biphenylene-like indium nitride (BPN-InN) via density functional theory and molecular dynamics simulations. The BPN-InN has a direct band gap energy transition of 2.02 eV, making it promising for optoelectronic applications. This structure exhibits maximum and minimum Young modulus of 22.716 and 22.063 N/m, Poisson ratio of 0.018 and −0.008, and Shear modulus of 11.448 and 10.860 N/m, respectively. To understand the BPN-InN behavior when subjected to mechanical deformations, biaxial and uniaxial strains in armchair and zigzag directions from −8 to 8% were applied, achieving a band gap energy modulation of 1.36 eV over tensile deformations. Our findings are expected to motivate both theorists and experimentalists to study and obtain these new 2D inorganic materials that exhibit promising semiconductor properties.

## 1 Introduction

The versatility of carbon in adopting sp, sp^2^, and sp^3^ hybridizations opens up an infinite array of crystalline lattices with a broad range of properties. Some examples are T-graphene,^1^ twin-graphene,^2^ QPHT-graphene,^3^ penta-graphene,^4^ DHP-graphene,^5^ TPDH-graphene,^6^ graphenylene,^7^ biphenylene,^8^ triphenylenes,^9^ naphthylenes,^10,11^ and tripentaphenes.^12,13^ In this structure class, biphenylene features a lattice composed of four-, six-, and eight-membered carbon rings, known as the 4-6-8-biphenylene network (BPN). Another related structure, graphenylene, is characterized by a lattice containing 4-, 6-, and 12-membered carbon rings. The first one was recently obtained by surface polymer dehydrogenation (HF-zipping),^14^ while the second one was produced via polymerization reactions using the 1,3,5-trihydroxybenene precursor.^15^ Many reports, mainly based on density functional theory (DFT), have demonstrated that both monolayers are suitable for diverse applications, including catalysis,^16^ molecular membranes,^7^ water purification,^17^ gas sensing,^18−20^ thermoelectricity,^21−23^ optoelectronics,^24^ energy storage,^25−27^ electronics,^28^ and catalysis.^29,30^

In the past decade, several inorganic graphenyelenes (IGPs) have been proposed based on diverse compounds such as boron nitride (BN),^31^ aluminum nitride (AlN),^32^ gallium nitride (GaN),^32^ indium nitride (InN),^33^ SiC,^34^ GeC,^35,36^ SiGe,^37^ ZnO,^38^ CdO,^39^ MgO,^39^ and BeO.^39^ Among them, IGP-ZnO showed up promising for SO_2_ and NO_2_ detection.^40^ At the same time, the IGP-SiC has proven itself to be an optimal platform for energy storage when employed in Na-based batteries^41^ and hydrogen storage.^42^ On the other hand, the same approach has been employed for two-dimensional (2D) biphenylene. The utilization of AlN and GaN as counterparts of the biphenylene network (BPN-AlN and BPN-GaN) led to two stable monolayers with band gap energies of 2.3 and 3.2 eV, respectively.^43^ Both monolayers exhibit noteworthy UV activity, promising prospects as UV collectors. Furthermore, BPN-BN has structural and dynamic stability, a direct band gap of 4.5 eV, and a Young modulus between 234.4 and 273.2 GPa.^44^ In the current year, four novel inorganic biphenylene lattices utilizing CdS and ZnS were introduced.^45^ These structures are stable at 300 K and belong to the class of ultrawideband gap semiconductors, featuring band gap energies ranging from 3.59 to 4.30 eV. Particularly noteworthy is the BPN-ZnS lattice, which displays a remarkable auxetic behavior.

In 2024, Laranjeira et al.^33^ proposed the IGP-InN, showing that this monolayer is stable at 700 K and exhibits a competitive cohesive energy compared to wurtzite InN. With a band gap energy of 2.49 eV, suitable for optoelectronic applications in the UV–visible range, its band gap can be adjusted by 1.19 eV under tensile strain. This discovery has led to the design of biphenylene-like InN (BPN-InN) as a viable alternative, leveraging the similarity between graphenylene and biphenylene lattices. Furthermore, free-standing 2D hexagonal indium nitride (h-InN) is a well-consolidated material with a variety of applications, including optoelectronics,^46,47^ gas sensing,^48^ and thermoelectrics.^49^

Based on the above observations, this report introduces, for the first time, the BPN-InN structure using density functional theory (DFT) simulations, showcasing the role of simulations in anticipating properties of unknown materials. A detailed study of its electronic, structural, mechanical, and vibrational properties was performed to characterize and demonstrate the potential of BPN-InN. The electronic analysis reveals that BPN-InN exhibits semiconductor characteristics, featuring a band gap energy of 2.02 eV, falling within the visible range. Furthermore, it has a structural buckling and a negative Poisson ratio, both uncommon traits within the BPN family. To comprehend the mechanical behavior of BPN-InN, biaxial and uniaxial strains were applied in the armchair and zigzag directions, ranging from −8 to 8%, resulting in a band gap energy modulation by 1.36 eV under tensile deformations.

## 2 Computational Setup

The computational simulations were carried out using the CRYSTAL17 package^50^ based on the DFT in combination with the HSE06 hybrid functional,^51,52^ and at the same time, the 9763111-631^53^ and 6-21G*^54^ basis sets were selected to represent the In and N atomic centers, respectively.

The precision of the infinite Coulomb and Hartree–Fock (HF) exchange series is controlled by five α*_i_* parameters with *i* = 1, 2, 3, 4, and 5, where α_1_ is the overlap, α_2_ is the penetration for Coulomb integrals, α_3_ is the overlap for HF exchange integrals and α_4_ and α_5_ are the pseudo-overlaps (HF exchange series). The two-electron contributions are neglected when the overlap between atomic functions is lower than 10^–α_*i*_^. For the calculations, the five α_*i*_ parameters were set to 20, 20, 20, 20, and 40, respectively. The convergence criterion for SCF is 10^–6^ au/cell, while for geometry optimization, it is 10^–7^ au/cell, and for elastic constant calculations, it is 10^–8^ au/cell. The optimization convergence was checked on the root-mean-square (RMS) and the absolute value of the largest component of both the gradients and the estimated displacements. The convergence criteria employed in the optimization for RMS and the largest component for gradient were 0.00030 and 0.00045 au and for displacement 0.00120 and 0.00180 au, respectively. The reciprocal space was sampled using Pack–Monkhorst and Gilat nets with sublattice and a shrinking factor of 6, resulting in 16 *k*-points in the irreducible Brillouin zone. The vibrational modes at the Γ point were evaluated using the numerical second derivatives of the total energies estimated with the coupled perturbed HF/Kohn–Sham (CPKS) algorithm.^55^

The quantum theory of atoms in molecules and crystals (QTAIMC)^56,57^ was employed to characterize the nature of chemical bonds. This approach uses the electronic density (ρ(*r*)) at the bond critical points (BCPs) to obtain topological parameters, such as the laplacian (∇^2^ρ(*r*)), the potential energy density (*V*(*r*)), the kinetic energy density (*G*(*r*)), and the total electronic energy density (*H*(*r*) = *V*(*r*) + *G*(*r*)). These parameters can provide valuable information regarding the type of bond interaction: shared electron pairs (shared shell or covalent bonds) or closed electron shells of one of the atoms (closed shell or ionic bonds).

The elastic constants (*C*_*ij*_) were calculated as the second derivative of the energy (*E*) concerning the strain component (ϵ_*i*_ and ϵ_*j*_) according to the following expression
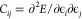
1

To analyze the anisotropic mechanical behavior of the structures presented herein, the orientation-dependent Young modulus *Y*(θ), Poisson ratio ν (θ), and Shear modulus *G*(θ) were calculated employing the following expressions^58^
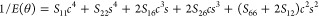
2
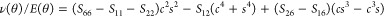
3
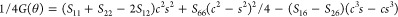
4where *s* = sin θ, *c* = cos θ, and θ ∈ [0, 2π] are the angle with respect to the +*x* axis. *S*_*ij*_ = *C*_*ij*_^–1^ are the elastic compliance constants.

The cohesive energy (*E*_coh_) was utilized to confirm the structural stability of the BPN-InN by the equation *E*_coh_ = (*E*_monolayer_ – *n*_In_*E*_In_ – *n*_N_*E*_N_)/(*n*_In_ + *n*_N_), where *E*_monolayer_ is the BPN-InN total energy, *E*_In_ and *E*_N_ are the energies of In and N isolated atoms, respectively, and *n*_In_ and *n*_N_ are the numbers of In and N atoms in the structure. For comparison purposes, *E*_coh_ was also calculated for h-InN (hexagonal 2D InN) and IGP-InN.

Molecular dynamics (MD) simulations were carried out using the extended tight-binding approximation (xTB)^59^ as implemented in the DFTB+ package,^60^ with the GFN1-xTB parametrization.^61^ The thermal stability of BPN-InN was performed using the Berendsen thermostat^62^ at 300 K by 4 ps with a time step of 1 fs. The rupture temperature of BPN-InN was obtained in a second way, increasing the temperature from 300 K up to the break employing the same time step.

## 3 Results and Discussion

**Figure 1:**
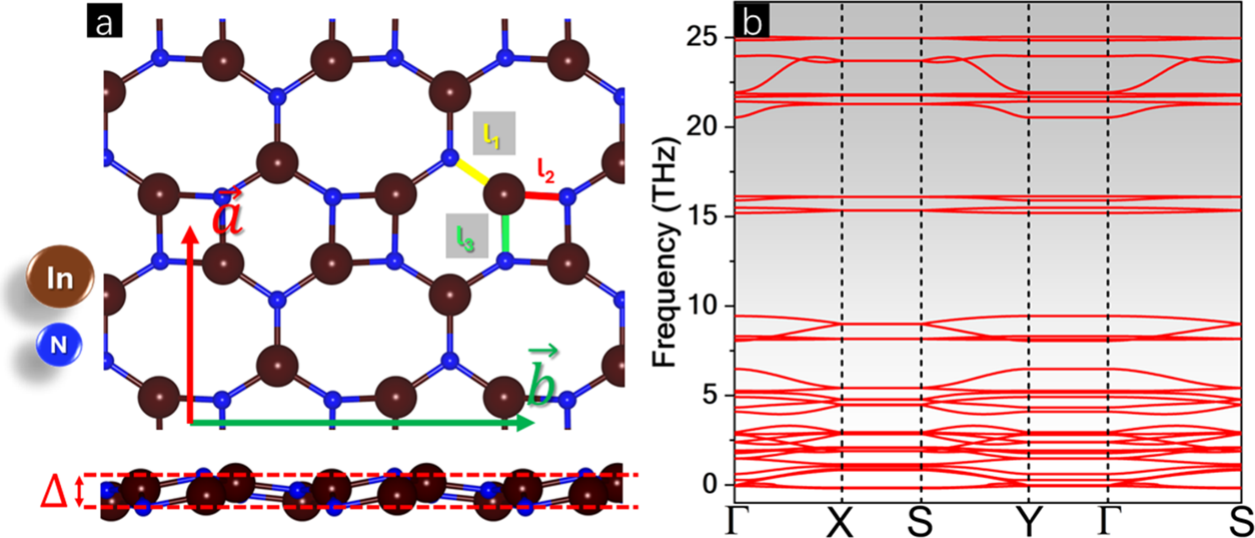
(a) Representation of BPN-InN with the lattice vectors, structural buckling (Δ), and nonequivalent bonds (*l*_1_, *l*_2_, and *l*_3_) and (b) phonon bands dispersion along the high-symmetry pathway in the Brillouin zone.

The BPN-InN structure has a rectangular buckled lattice (Δ) of 1.04 Å, belongs to the space group *Pm* (no. 6) with lattice parameters *a* = 6.34 Å, *b* = 10.81 Å, and α = β = 90° and possesses three different bond lengths, namely, *l*_1_ = 2.04 Å, *l*_2_ = 2.07 Å, and *l*_3_ = 2.12 Å, as represented in Figure 1. The octagonal pore has a maximum diameter of 5.73 Å. The unit cell contains eight nonequivalent atoms, and its internal coordinates are detailed in the crystallographic information file (CIF) in the Supporting Information (SI). The cohesive energy of BPN-InN is −4.29 eV/atom, a value closer than the obtained for IGP-InN (−4.22 eV/atom). The magnitude of the cohesive energy demonstrates the viability of BPN-InN from an energetic point of view.

The phonon dispersion along the high-symmetry pathways was calculated to analyze the dynamical stability, as displayed in Figure 1b. Two negative phonon modes with a minimum frequency of −0.18 THz (−5 cm^–1^) are noticed. Such small imaginary frequencies could be an artifact of limited supercell size considered (4 × 4 × 1 supercell), *k*-points, or reflect the lattice instability over large wave undulations. Wang et al.^58^ define −2 THz in the phonon vibration frequency as the limit for free-standing monolayers. Nevertheless, in phonon dispersion bands, it can be verified that the bands are generally flat, which denotes lower phonon propagation. At Γ and *Y* points, a quadradic dispersion is noticed, and two phononic band gaps occur (10–15 and 16–20 THz). These band gaps allow phonon propagation control, tailoring the thermal and acoustic properties for applications such as thermal insulators, phononic crystals, and energy harvesting and conversion.

**Figure 2:**
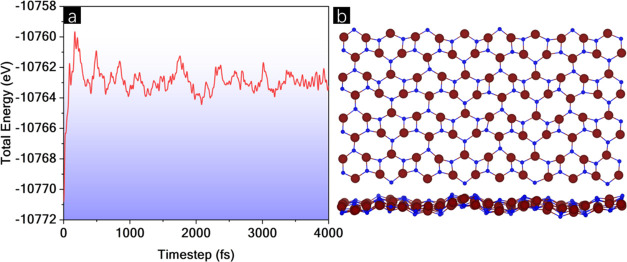
(a) Total energy profile along thermal stability (at 300 K) molecular dynamics simulations and (b) last iteration snapshot.

Thermal stability was carried out from 300 K by 4 ps, as shown in Figure 2, which offers a top and side view of the last interaction in MD simulations with a 4 × 4 × 1 supercell with 192 atoms. It can be observed that the total energy remains in a flat pattern, with energy fluctuations in the order of meV/atom. The MD snapshot revealed that the BPN-InN monolayer maintains its ring structure and does not undergo bond reconstruction during the simulations. The rupture temperature of BPN-InN was determined by gradually increasing the temperature, using a fixed time step of 1 fs in the heating simulation. The simulations indicate that BPN-InN is stable up to approximately 920 K, as no bond breakages or reconstructions were observed. A video animation of the MD simulations is available in the SI.

**Figure 3:**
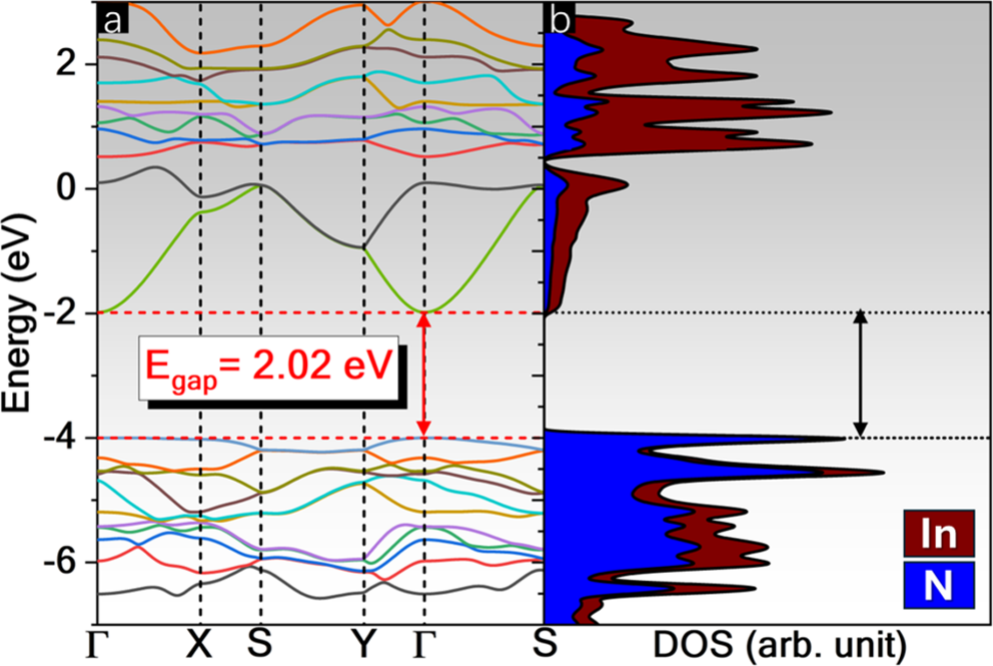
(a) Band structure and (b) density of states of BPN-InN.

The band structure and density of states (DOS) of BPN-InN, shown in Figure 3, were analyzed to study the band dispersion and electronic state distribution. A direct band gap transition at Γ point is observed with an energy (*E*_gap_) of 2.02 eV. The BPN-InN exhibits the lowest band gap when compared to the other BPN-based nitrides, namely, BPN-AlN, BPN-GaN,^43^ and BPN-BN,^44^ with band gap energies of 2.30, 3.18, and 4.50 eV, respectively. In the class of the III–IV BPN analogs, all reported via DFT simulations, only the InN and BN-based possess direct band gap transition. Their low *E*_gap_ value and direct transition make the BPN-InN the most promising for optoelectronic applications.

Furthermore, it is interesting to note that in BPN-InN, the valence band maximum (VBM) has a flatness aspect, characterizing heavier photogenerated holes. At the same time, the conduction band minimum (CBM) denotes a parabolic-like format that indicates lighter electrons. These features indicate a lower recombination ratio, which is an essential requisite for the photocatalytic process. The suitability of BPN-InN in photocatalysis is corroborated by its visible range *E*_gap_.

The DOS reveals significant contributions from In states in both the conduction and valence bands within the energy interval considered. At the VBM, there is a higher contribution from N atoms, which significantly decreases in the CBM. The edge regions of the DOS support the band structure analysis. The VBM shows a high number of states, which drop abruptly to zero in the band gap, indicating highly correlated states and lower carrier mobility. Conversely, the CBM has a low DOS, indicating high band dispersion and, consequently, higher carrier mobility.

The results of the topological analysis are presented in Table 1. The ∇^2^ρ(*r*) values, which are close to zero and positive, indicate transient bonds that bridge the gap between covalent and ionic interactions. A |*V*(*r*)|/*G*(*r*) ratio lower than 1 for all bonds suggests ionic interactions, while negative *H*/ρ(*r*) values indicate covalent bonds. This analysis shows that the In–N bonds cannot be strictly classified as covalent or ionic, as they lie between shared and closed-shell interactions. Notably, the *l*_1_, *l*_2_, and *l*_3_ values are very close for all parameters, indicating nearly identical bonds from a topological perspective.

**Table 1:** Topological Parameters Based on the QTAIMC Analysis for *l*_1_, *l*_2_, and *l*_3_ Bonds in the BPN-InN^a^

bond	ρ(*r*)	∇^2^ρ(*r*)	|*V*(*r*)|/*G*(*r*)	*H*/ρ(*r*)
*l*_1_	0.091	0.321	0.121	–0.238
*l*_2_	0.098	0.358	0.121	–0.241
*l*_3_	0.090	0.320	0.121	–0.238

a Where ρ(*r*) is the charge density, ∇^2^ρ(*r*) is the Laplacian of the charge density, |*V*(*r*)|/*G*(*r*) is the ratio between the virial (*V*(*r*)) and the kinetic density energy (*G*(*r*)), and (*H*(*r*)/ρ(*r*)) is the bond degree all calculated on the bond critical points (BCPs).

**Figure 4:**
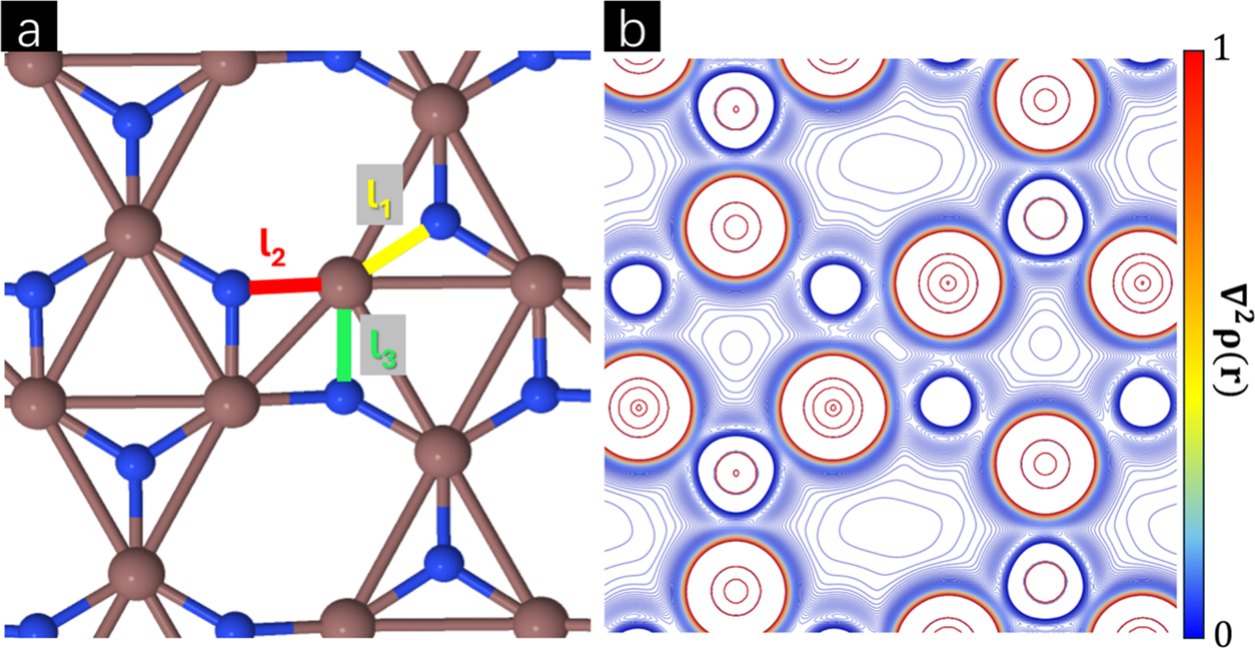
(a) BPN-InN and its nonequivalent bonds, *l*_1_, *l*_2_, and *l*_3_, and (b) electronic density laplacian (∇^2^ρ(*r*)).

The electronic density Laplacian map was plotted as shown in Figure 4. This representation allows us to understand how the electronic density varies around the BCPs. As corroborated by Table 1, small positive values for ∇^2^ρ(*r*) can be observed between the bonds, indicating interactions with a weak shared-shell character. Due to the planar representation, the *l*_2_ and *l*_3_ bonds appear to have lower ∇^2^ρ(*r*) values compared to *l*_1_. The absence of ∇^2^ρ(*r*) isolines reveals charge depletion centers in the N atoms, while the opposite behavior indicates charge accumulation centers in the In atoms. When interacting with other species, the N can act as an electron donor and In as a receptor.

**Figure 5:**
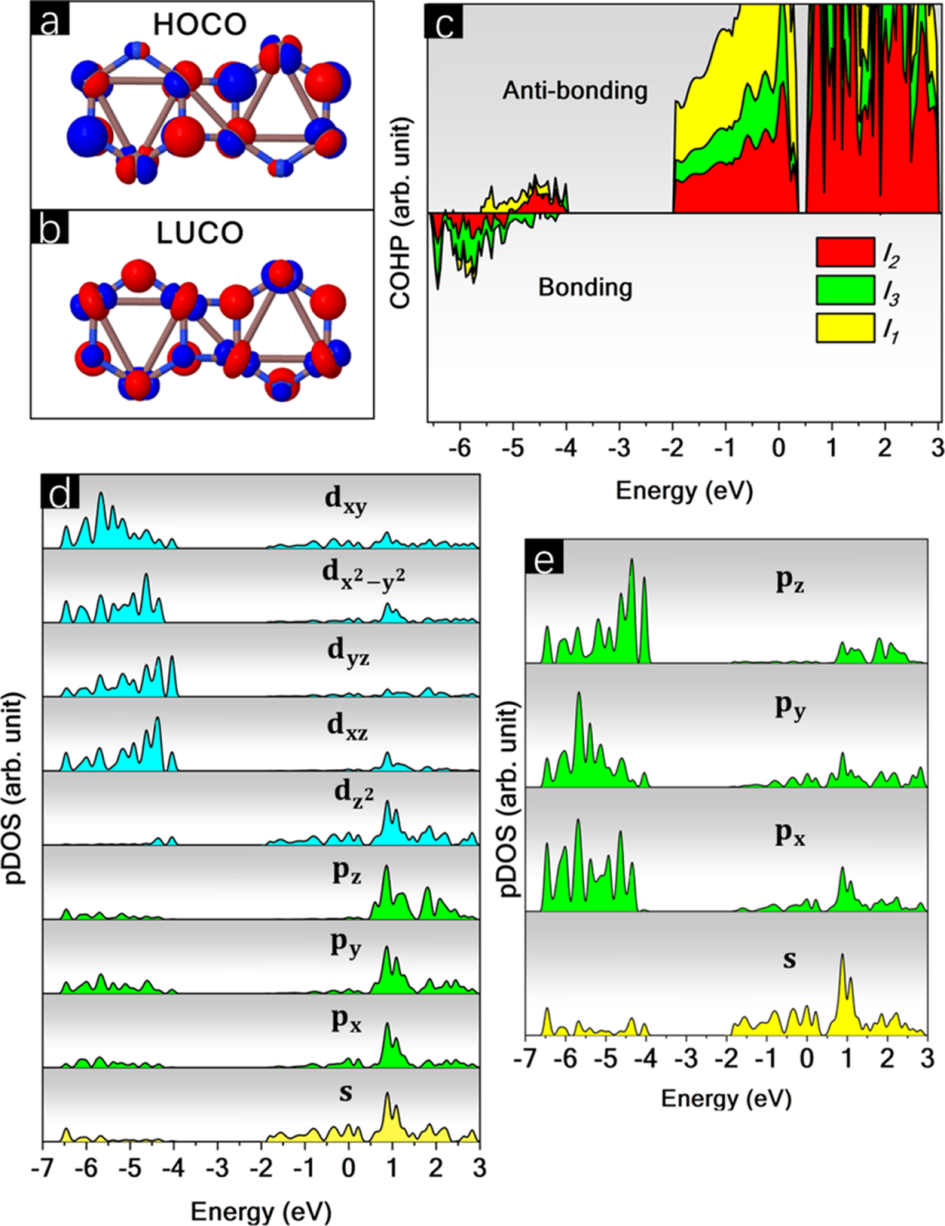
(a) Highest occupied crystalline orbital (HOCO), (b) lowest unoccupied crystalline orbital (LUCO), (c) crystal orbital Hamilton population (COHP), and density of states per orbital (pDOS) for (d) In, and (e) N.

Figure 5 represents the highest occupied crystalline orbital (HOCO) and lowest unoccupied crystalline orbital (LUCO), crystal orbital Hamilton population (COHP) for all *l*_1_, *l*_2_, and *l*_3_ bonds, and the density of states per orbital (pDOS) for In and N.

First, the absence of orbital overlap for both HOCO and LUCO, as corroborated by the COHP, indicates that all bonds are antibonding in the VBM and CBM.

Analyzing the pDOS for In, the major contributions in the VBM mainly come from the d_*xz*_ and d_*yz*_ orbitals. On the other hand, the CBM is predominantly composed of d_*z*^2^_ and s orbitals. Regarding the In_d_ orbitals, the pDOS indicates a breakdown in degeneracy, resulting in higher energy for d orbitals with a *z* component. This finding can be related to the high buckling reported for BPN-InN, which emerges as a result of sp^2^–sp^3^ mixed bonds.

The pDOS for N atoms corroborates the analysis performed for In, demonstrating total degeneracy splitting, with the *p*_*z*_ orbitals predominating in the VBM. Due to symmetry considerations, it is expected that bonds with higher σ characters. This is in agreement with the topological parameters, which show a lower ρ(*r*) and ∇^2^ρ(*r*) at the bond critical points (BCPs), indicating a lower orbital overlap from the perspective of a purely covalent bond.

The mechanical properties of BPN-InN were analyzed, and the results are displayed in Table 2. The calculated BPN-InN elastic constants are *C*_11_ = 22.07 N/m, *C*_12_ = 0.41 N/m, *C*_22_ = 22.18 N/m, and *C*_66_ = 11.45 N/m. The Born–Huang criteria^63^ plays a crucial role in determining the mechanical stability of 2D materials. For rectangular lattices, the key conditions, namely *C*_11_ > 0, *C*_66_ > 0, and *C*_11_*C*_22_ > (*C*_12_)^2^, were fulfilled, confirming the mechanical stability of BPN-InN. BPN-InN exhibits maximum and minimum Young modulus (*Y*_max_ and *Y*_min_, respectively) of 22.716 and 22.063 N/m, Poisson ratio (ν_max_ and ν_min_, respectively) of 0.018 and −0.008, and shear modulus (*G*_max_ and *G*_min_, respectively) of 11.448 and 10.860 N/m.

**Table 2:** Elastic Constants (*C*_11_, *C*_12_, *C*_22_, *C*_66_) (N/m), Maximum and Minimum Values of Young’s Modulus (*Y*_max_, *Y*_min_) (N/m), Poisson’s Ratio (ν_max_, ν_min_), and Shear Modulus (*G*_max_, *G*_min_) (N/m) for BPN-InN, IGP-InN, and h-InN (HSE06/DFT, Superscript a), along with Biphenylene-Like Inorganic Monolayers from References (43) (Superscript b) and (39) (Superscript c)

	*C*_11_	*C*_12_	*C*_22_	*C*_66_	*Y*_max_/*Y*_min_	ν_max_/ν_min_	*G*_max_/*G*_min_
BPN-InN^a^	22.07	0.41	22.18	11.45	22.716/22.063	–0.008/0.018	11.448/10.860
IGP-InN^a^	15.41	0.46	15.41	7.48	15.400/15.400	0.030/0.030	7.479/7.479
h-InN^a^	119.63	69.91	119.63	119.63	78.772/78.772	0.580/0.580	24.878/24.859
BPN-GaN^b^	124.51	33.47	104.73		114.50/–	0.82/0.11	
BPN-AlN^b^	172.93	45.44	135.97		158.39/–	0.91/0.20	
BPN-BeO^c^					115.00/78.09	0.54/0.35	
BPN-MgO^c^					60.83/29.57	0.87/0.43	
BPN-CdO^c^					32.11/16.67	1.00/0.57	
BPN-ZnO^c^					52.47/29.89	0.90/0.54	
IGP-BeO^c^					97.96	0.33	
IGP-MgO^c^					54.35	0.45	
IGP-CdO^c^					28.48	0.59	
IGP-ZnO^c^					48.08	0.55	

**Figure 6:**
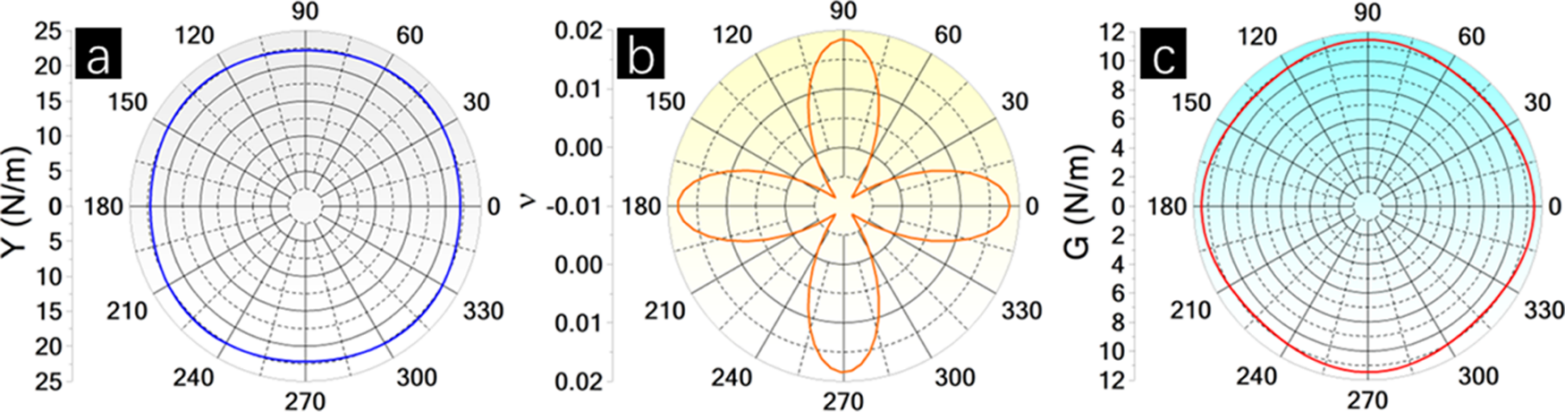
Polar diagrams for (a) Young modulus (*Y*), (b) Poisson ratio (v), and (c) Shear modulus (*G*) of BPN-InN.

The anisotropy is more remarkable for the Poisson ratio, as seen in the polar diagrams in Figure 6. The Young and Shear moduli show a circular-like distribution and, therefore, reduced anisotropy. In comparison with its analogs, i.e., IGP-InN and h-InN, the anisotropy in ν on BPN-InN is a distinctive characteristic considering the almost perfectly isotropic behavior of the two first relative to *Y*, ν, and *G*. Concerning the *Y*_max_ and *G*_max_, the BPN-InN is more rigid than IGP-InN and significantly softer than h-InN.

Poisson ratio (ν) measures the ratio of lateral strain to axial strain in a material. A Poisson ratio of precisely zero would imply that the material does not change in lateral dimensions when stretched or compressed, which is a rare and idealized case. Interestingly, both BPN-InN and IGP-InN exhibit ν closer to zero, which is not reported for other inorganic BPN and IGP-based structures. For example, BPN-AlN and BPN-GaN possess ν_max_ = 0.91 and 0.82 and ν_min_ = 0.20 and 0.11, respectively.^43^ Abdullahi and Ersan reported ν_max_ = 0.54, 0.87, 0.90, and 1.00 and ν_min_ = 0.35, 0.43, 0.54, and 0.54 for BPN-BeO, BPN-MgO, BPN-ZnO, and BPN-CdO, respectively.^39^ In the same work, the authors also provide ν = 0.33, 0.45, 0.55, and 0.59 for IGP-BeO, IGP-MgO, IGP-ZnO, and IGP-CdO. Fabris et al.^32^ showed ν = 0.38 for IGP-GaN, and Martins et al., ν = 0.33 for IGP-GeC.^35^ On the other hand, h-InN exhibits higher ν (0.58), as verified by Peng et al.^64^ that obtained ν = 0.59. These findings demonstrate that, in particular, InN biphenylene-based structures result in a closer to zero Poisson ratio.

**Figure 7:**
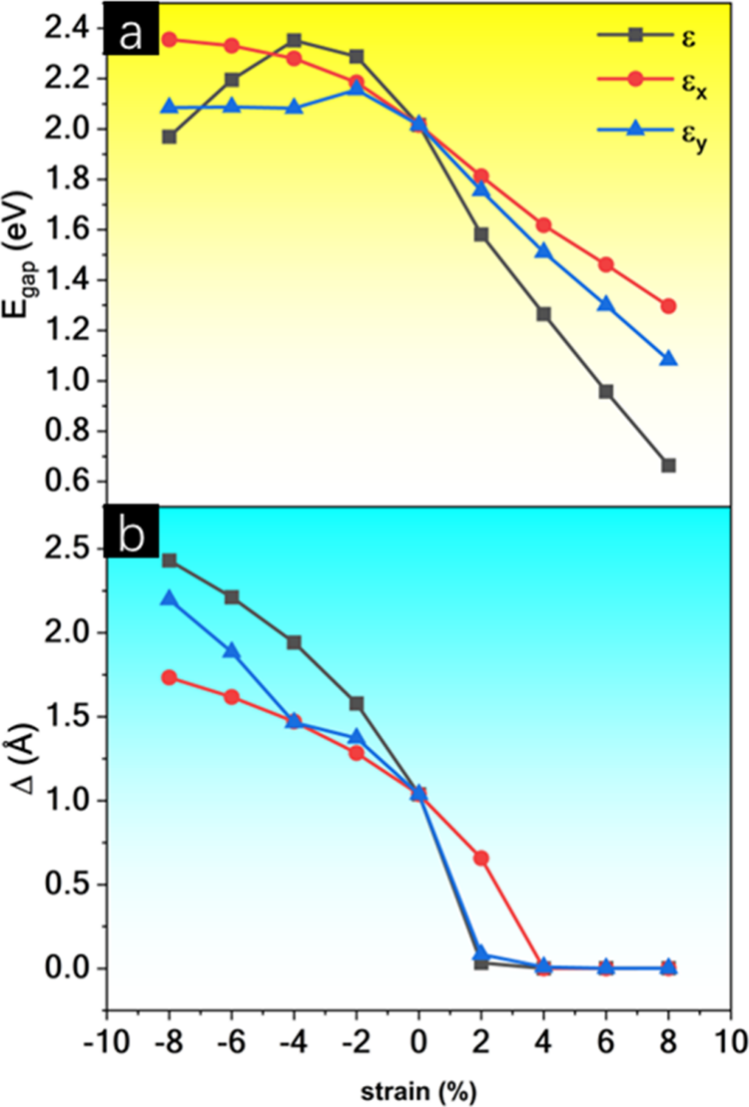
(a) Band gap energy and (b) buckling (Δ) as functions of biaxial (ε) and uniaxial (ε_*x*_ and ε_*y*_) strains.

To investigate the behavior of BPN-InN under mechanical deformations, biaxial strains (ε) as well as uniaxial strains in the armchair (ε_*x*_), and zigzag (ε_*y*_) directions ranging from −8 to 8% were applied to the monolayer. Figure 7 shows the *E*_gap_ and the buckling (Δ) as functions of the strain. For tensile strains, i.e., positive ε, ε_*x*_, and ε_*y*_ values, a notable decrease of *E*_gap_ is verified for BPN-InN. The biaxial strain reinforces this decrease, while when uniaxial strains act, it is softened.

The *E*_gap_ achieved their minimum for ε = 8% (*E*_gap_ = 0.66 eV) and the maximum for ε_*x*_ = −8% (*E*_gap_ = 2.36 eV). A variation of 1.36 eV is noticed for tensile ε in the *E*_gap_, greater than the 1.19 eV reported by Laranjeira et al.^33^ for IGP-InN at WC1LYP/DFT level. In this sense, Li et al.^65^ showed a decrease of 1.20 eV for the MoS_2_ monolayer for biaxial tensile strains. Mortazavi and Rabczuk^66^ achieved *E*_gap_ decreases of 1.02, 0.95, 0.99, and 1.32 eV for SiP, SiAs, GeP, and GeAs monolayers, respectively. These findings show that strain engineering is a suitable strategy to fine-tune the electronic properties of BPN-InN.

The buckling (Δ) increases under compressive strains and decreases under tensile strains. The Δ increase is higher for biaxial strains than for unaxial. Buckling reaches its maximum value at ε = −8% (2.43 Å) and decreases until it disappears at ε = ε_*x*_ = ε_*y*_ = 4%. Beyond 4%, Δ it remains zero. Li et al.^67^ reported a similar phenomenon for penta-B_2_C, observing buckling values of 2.24 and 0.00 Å for ε = −20 and 20%, respectively. This indicates that planarity can be achieved in BPN-InN with significantly lower deformations compared to penta-B_2_C. For IGP-InN employing the same strain range, a buckling variation of approximately 2.6 Å was observed, consistent with the behavior of BPN-InN. Importantly, when BPN-InN becomes planar, a new symmetry emerges, indicating a phase transition induced by mechanical strain. Under biaxial strain, this new structure belongs to space group *Pmma* (no. 51) with lattice parameters *a* = 11.028 Å and *b* = 6.467 Å. It contains four nonequivalent atoms, as detailed in the CIF file in the SI. Similarly, Laranjeira et al.^33^ observed that tensile strains in IGP-InN (space group *P*3, no. 147) decrease the buckling to zero, resulting in a planar structure with increased symmetry (space group *P*6/*m*, no. 175).

**Figure 8:**
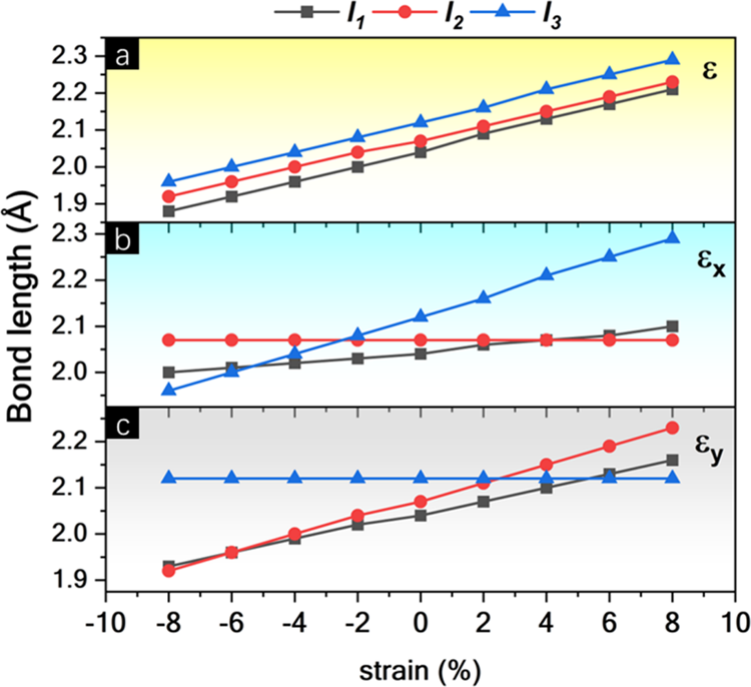
Bond lengths (*l*_1_, *l*_2_, and *l*_3_) as functions of (a) biaxial (ε), (b) armchair (ε_*x*_), and (c) zigzag (ε_*y*_) strains.

From the perspective of bond lengths, as shown in Figure 8, when subjected to biaxial strain, the BPN-InN bonds (*l*_1_, *l*_2_, and *l*_3_) exhibit approximately equal behavior, with all *l*_1_, *l*_2_, and *l*_3_ describing linear curves with similar inclination concerning. This result is consistent with the topological analysis, which showed that the *l*_1_, *l*_2_, and *l*_3_ bonds are practically identical.

When evaluating the armchair deformations ε_*x*_, the *l*_1_ and *l*_2_ bonds do not show significant modifications, while the *l*_3_ bond distributes the tension along the lattice. In contrast, for ε_*y*_, an inverse behavior to ε_*x*_ is observed, with *l*_1_ and *l*_2_ being most influenced by the strain, while *l*_3_ remains constant.

**Figure 9:**
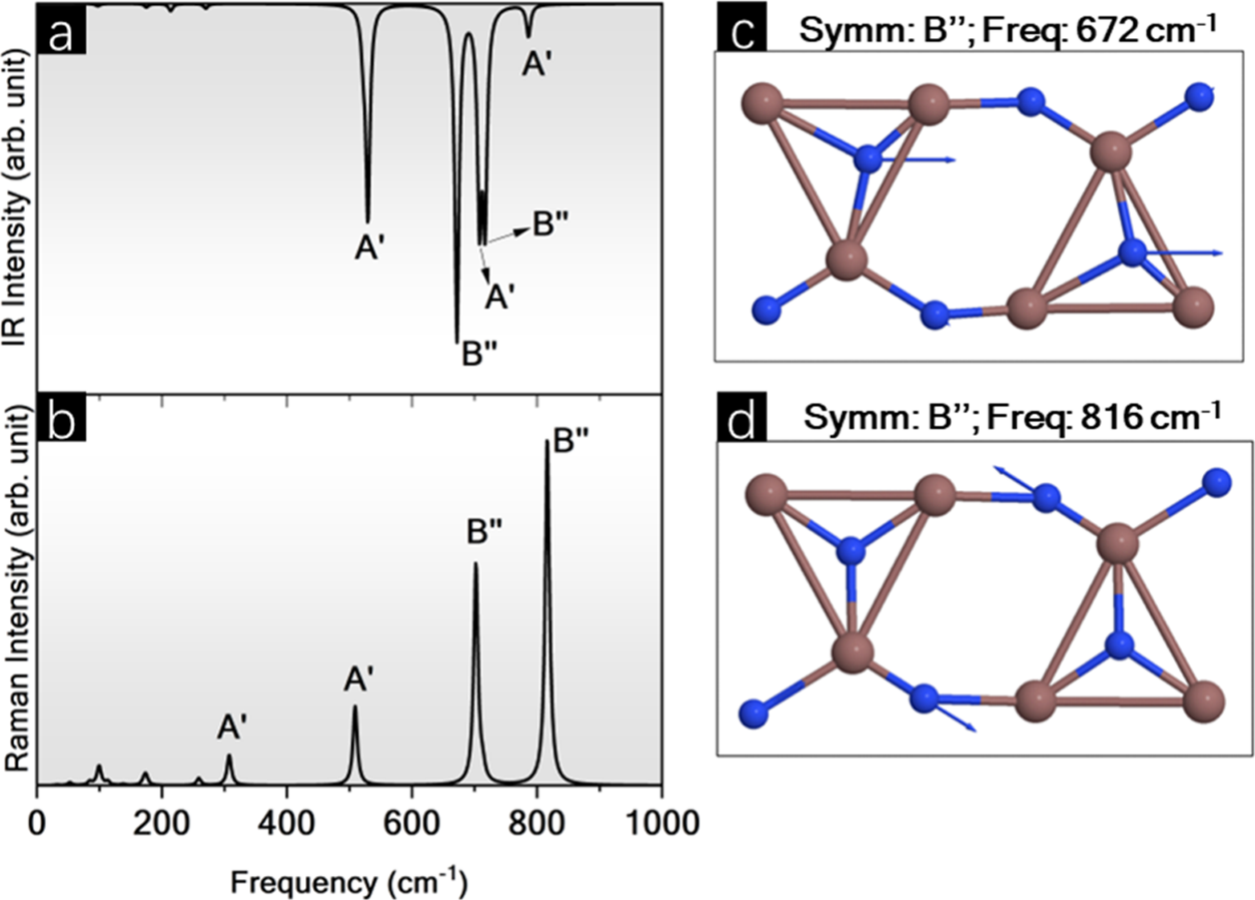
(a) Raman and (b) Infrared (IR) spectra obtained through the CPKS approach, along with corresponding vibrational modes for the most intense peaks in (c) IR and (d) Raman.

The vibrational analysis was performed via the coupled perturbed Kohn–Sham (CPKS) algorithm^68^ to evaluate the short-range order in BPN-InN. It revealed the existence of 36 vibrational active modes Γ_vib_ = 20A′ + 16B″, all active in both Raman and infrared (IR). The Raman and IR spectra are represented in Figure 9. For IR (Figure 9a), the highest peak occurs at 672 cm^–1^ with B″ symmetry that corresponds to symmetric stretching in the *l*_1_, *l*_2_, and *l*_3_ bonds (Figure 9c). Conversely, the Raman spectrum (Figure 9b) shows the largest peak at 816 cm^–1^ related to asymmetric stretching of *l*_1_ and *l*_3_ bonds (Figure 9d). The peaks highlighted for the IR spectrum occur at 529 cm^–1^ (A′), 672 cm^–1^ (B″), 708 cm^–1^ (A′), 717 cm^–1^ (A′), and 786 cm^–1^ (A′). For the Raman spectrum are highlighted the peaks at 308 cm^–1^ (A′), 509 cm^–1^ (A′), 702 cm^–1^ (B″), and 816 cm^–1^ (B″).

## 4 Conclusions

In this work, DFT/HSE06 simulations were performed to characterize the novel biphenylene-like InN in terms of its structural, electronic, mechanical, and vibrational properties. Molecular dynamics demonstrated that this monolayer is stable up to 920 K without reconstructions and bond breakages. Phonon dispersion bands reveal small imaginary modes that are also discussed in the literature and that do not denote the instability of the material. The electronic analysis showed a direct band gap transition of 2.02 eV, promising for visible-range optoelectronic applications. The contribution of the In states is greater than N in both the conduction and valence bands. From the mechanical properties, it was noticed that BPN-InN fulfills the Born–Huang criteria. The Young modulus, Poisson ratio, and shear modulus ranged values of 22.716 and 22.063 N/m, 0.018 and -0.008, and 11.448 and 10.860 N/m, respectively. Biaxial and uniaxial strains in armchair and zigzag directions from −8 to 8% were applied, and a phase transition induced by strain was observed when the structure became utterly planar. An expressive band gap modulation of 1.36 eV over tensile deformations was reported. Our findings are expected to motivate both theorists and experimentalists to explore and synthesize biphenylene-based InN structures, which exhibit promising semiconductor properties as demonstrated herein.

## Supporting Information Available

Video referring to heating molecular dynamics simulation from 300 K up to rupture (MP4)
Crystallographic information file of pristine BPN-InN (CIF)
Crystallographic information file of planar BPN-InN (CIF)

## Supplementary Material

ao4c03511_si_001.mp4

ao4c03511_si_002.cif

ao4c03511_si_003.cif

## References

[ref1] Liu Y.; Wang G.; Huang Q.; Guo L.; Chen X. Structural and electronic properties of T graphene: A two-dimensional carbon allotrope with tetrarings. Phys. Rev. Lett. 2012, 108, 225505 10.1103/PhysRevLett.108.225505. 23003621

[ref2] Jiang J. W.; Leng J.; Li J.; Guo Z.; Chang T.; Guo X.; Zhang T. Twin graphene: A novel two-dimensional semiconducting carbon allotrope. Carbon 2017, 118, 370–375. 10.1016/j.carbon.2017.03.067.

[ref3] Wang X.; Rong J.; Song Y.; Yu X.; Zhan Z.; Deng J. QPHT-graphene: A new two-dimensional metallic carbon allotrope. Phys. Lett. A 2017, 381, 2845–2849. 10.1016/j.physleta.2017.06.035.

[ref4] Zhang S.; Zhou J.; Wang Q.; Chen X.; Kawazoe Y.; Jena P. Penta-graphene: A new carbon allotrope. Proc. Natl. Acad. Sci. U.S.A. 2015, 112, 2372–2377. 10.1073/pnas.1416591112. 25646451 PMC4345574

[ref5] Cai Y.; Guo Y.; Jiang B.; Lv Y. Encapsulation of cathode in lithium-sulfur batteries with a novel two-dimensional carbon allotrope: DHP-graphene. Sci. Rep. 2017, 7, 14948 10.1038/s41598-017-15010-7. 29097737 PMC5668302

[ref6] Bhattacharya D.; Jana D. TPDH-graphene: A new two dimensional metallic carbon with NDR behaviour of its one dimensional derivatives. Phys. E 2021, 127, 114569 10.1016/j.physe.2020.114569.

[ref7] Song Q.; Wang B.; Deng K.; Feng X.; Wagner M.; Gale J. D.; et al. Graphenylene, a unique two-dimensional carbon network with nondelocalized cyclohexatriene units. J. Mater. Chem. C 2013, 1, 38–41. 10.1039/C2TC00006G.

[ref8] Hudspeth M. A.; Whitman B. W.; Barone V.; Peralta J. E. Electronic properties of the biphenylene sheet and its one-dimensional derivatives. ACS Nano 2010, 4, 4565–4570. 10.1021/nn100758h. 20669980

[ref9] Dos Santos M. R.; Silva P. V.; Meunier V.; Girão E. C. Electronic properties of 2D and 1D carbon allotropes based on a triphenylene structural unit. Phys. Chem. Chem. Phys. 2021, 23, 25114–25125. 10.1039/D1CP00816A. 34714315

[ref10] Beserra D. J. P.; Saraiva-Souza A.; Diniz E. M.; Fadel M.; Meunier V.; Girão E. C. Naphthylene- γ: 1D and 2D carbon allotropes based on the fusion of phenyl- And naphthyl-like groups. Phys. Rev. Mater. 2020, 4, 084003 10.1103/PhysRevMaterials.4.084003.

[ref11] Álvares Paz M. L.; Saraiva-Souza A.; Meunier V.; Girão E. C. Naphthylenes: 1D and 2D carbon allotropes based on naphthyl units. Carbon 2019, 153, 792–803. 10.1016/j.carbon.2019.07.037.

[ref12] Silva P. V.; Fadel M.; Souza Filho A. G.; Meunier V.; Girão E. C. Tripentaphenes: two-dimensional acepentalene-based nanocarbon allotropes. Phys. Chem. Chem. Phys. 2020, 22, 23195–23206. 10.1039/D0CP02574G. 33026379

[ref13] Zeng T.; Yang H.; Wang H.; Chen G. Acepentalene Membrane Sheet: A Metallic Two-Dimensional Carbon Allotrope with High Carrier Mobility for Lithium Ion Battery Anodes. J. Phys. Chem. C 2020, 124, 5999–6011. 10.1021/ACS.JPCC.0C00376.

[ref14] Fan Q.; Yan L.; Tripp M. W.; Krejčí O.; Dimosthenous S.; Kachel S. R.; et al. Biphenylene network: A nonbenzenoid carbon allotrope. Science 2021, 372, 852–856. 10.1126/science.abg4509. 34016779

[ref15] Du Q. S.; Tang P. D.; Huang H. L.; Du F. L.; Huang K.; Xie N. Z.; et al. A new type of two-dimensional carbon crystal prepared from 1,3,5-trihydroxybenzene. Sci. Rep. 2017, 7, 40796 10.1038/srep40796. 28094298 PMC5240129

[ref16] Tang Y.; Chen W.; Zhang H.; Wang Z.; Teng D.; Cui Y.; et al. Single-atom metal-modified graphenylene as a high-activity catalyst for CO and NO oxidation. Phys. Chem. Chem. Phys. 2020, 22, 16224–16235. 10.1039/D0CP01062F. 32643727

[ref17] Jahangirzadeh M.; Azamat J.; Erfan-Niya H. Molecular insight into water desalination through functionalized graphenylene nanosheet membranes. Comput. Mater. Sci. 2022, 203, 111126 10.1016/j.commatsci.2021.111126.

[ref18] Saadh M. J.; Jasim S. A.; Veloz M. G.; Kumar A.; Mekkey S. M.; Guadalupe M. A.; et al. Evaluating ammonia sensors based on two-dimensional pure and silicon-decorated biphenylene using DFT calculations. Inorg. Chem. Commun. 2024, 160, 111918 10.1016/j.inoche.2023.111918.

[ref19] Mahamiya V.; Dewangan J.; Shukla A.; Chakraborty B. Remarkable enhancement in catechol sensing by the decoration of selective transition metals in biphenylene sheet: A systematic first-principles study. J. Phys. D: Appl. Phys. 2022, 55, 505401 10.1088/1361-6463/ac9ac3.

[ref20] Hosseini M. R.; Esfandiarpour R.; Taghipour S.; Badalkhani-Khamseh F. Theoretical study on the Al-doped biphenylene nanosheets as NO sensors. Chem. Phys. Lett. 2020, 754, 137712 10.1016/j.cplett.2020.137712.

[ref21] Zhang P.; Ouyang T.; Tang C.; He C.; Li J.; Zhang C.; et al. The intrinsic thermal transport properties of the biphenylene network and the influence of hydrogenation: a first-principles study. J. Mater. Chem. C 2021, 9, 16945–16951. 10.1039/D1TC04154A.

[ref22] Kumar A.; Senapati P.; Parida P. Theoretical insights into the structural, electronic and thermoelectric properties of the inorganic biphenylene monolayer. Phys. Chem. Chem. Phys. 2024, 26, 2044 10.1039/D3CP03088A. 38126442

[ref23] Xie Z. X.; Chen X. K.; Yu X.; Deng Y. X.; Zhang Y.; Zhou W. X.; Jia P. Z. Intrinsic thermoelectric properties in biphenylene nanoribbons and effect of lattice defects. Comput. Mater. Sci. 2023, 220, 112041 10.1016/j.commatsci.2023.112041.

[ref24] Demirci S.; Gorkan T.; Çallıoğlu Ş.; Çallloğlu Ş.; Özçelik V. O.; Barth J. V.; Aktürk E. Hydrogenated Carbon Monolayer in Biphenylene Network Offers a Potential Paradigm for Nanoelectronic Devices. J. Phys. Chem. C 2022, 126, 15491–15500. 10.1021/ACS.JPCC.2C04453.

[ref25] Mahamiya V.; Shukla A.; Chakraborty B. Ultrahigh reversible hydrogen storage in K and Ca decorated 4–6-8 biphenylene sheet. Int. J. Hydrogen Energy 2022, 47, 41833–41847. 10.1016/j.ijhydene.2022.01.216.

[ref26] Denis P. A.; Iribarne F. Hydrogen storage in doped biphenylene based sheets. Comput. Theor. Chem. 2015, 1062, 30–35. 10.1016/j.comptc.2015.03.012.

[ref27] Jiang J.; Chen Y.; Guo H.; Wu X.; Lu N.; Zhuo Z. Two-Dimensional Biphenylene-Based Carbon Allotrope Family with High Potassium Storage Ability. J. Phys. Chem. Lett. 2023, 14, 9655–9664. 10.1021/acs.jpclett.3c02396. 37870573

[ref28] Liu G.; Chen T.; Li X.; Xu Z.; Xiao X. Electronic transport in biphenylene network monolayer: Proposals for 2D multifunctional carbon-based nanodevices. Appl. Surf. Sci. 2022, 599, 153993 10.1016/j.apsusc.2022.153993.

[ref29] Singh M.; Shukla A.; Chakraborty B. Improving hydrogen evolution catalytic activity of 2D carbon allotrope biphenylene with B, N, P doping: Density functional theory investigations. Int. J. Hydrogen Energy 2024, 52, 569–579. 10.1016/j.ijhydene.2023.08.359.

[ref30] Luo Y.; Ren C.; Xu Y.; Yu J.; Wang S.; Sun M. A first principles investigation on the structural, mechanical, electronic, and catalytic properties of biphenylene. Sci. Rep. 2021, 11, 19008 10.1038/s41598-021-98261-9. 34561479 PMC8463688

[ref31] Fabris G. S. L.; Marana N. L.; Longo E.; Sambrano J. R. Theoretical study of porous surfaces derived from graphene and boron nitride. J. Solid State Chem. 2018, 258, 247–255. 10.1016/j.jssc.2017.10.025.

[ref32] Fabris G. S. L.; Paskocimas C. A.; Sambrano J. R.; Paupitz R. One- and two-dimensional structures based on gallium nitride. J. Solid State Chem. 2021, 303, 122513 10.1016/j.jssc.2021.122513.

[ref33] Laranjeira J. A. S.; Silva J. F.; Denis P. A.; Maia A. S.; Sambrano J. R. Novel buckled graphenylene-like InN and its strain engineering effects. Comput. Theor. Chem. 2024, 1231, 114418 10.1016/j.comptc.2023.114418.

[ref34] Martins N. F.; Fabris G. S. L.; Albuquerque A. R.; Sambrano J. R. A new multifunctional two-dimensional monolayer based on silicon carbide. FlatChem 2021, 30, 100286 10.1016/j.flatc.2021.100286.

[ref35] Martins N. F.; Laranjeira J. A. S.; Azevedo S. A.; Fabris G. S. L.; Sambrano J. R. Structural, electronic and mechanical properties of a novel graphenylene-like structure based on GeC. J. Phys. Chem. Solids 2023, 181, 111518 10.1016/j.jpcs.2023.111518.

[ref36] Abdullahi Y. Z.; Ersan F. Theoretical design of porous dodecagonal germanium carbide (d-GeC) monolayer. RSC Adv. 2023, 13, 3290–3294. 10.1039/D2RA07841D. 36756449 PMC9869739

[ref37] Kremer L. F.; Baierle R. J. Stability, electronic and optical properties of group IV graphenylene-like materials. An ab initio investigation. Diamond Relat. Mater. 2024, 141, 110689 10.1016/j.diamond.2023.110689.

[ref38] Fabris G. S. L.; Marana N. L.; Laranjeira J. A. S.; Longo E.; Sambrano J. R. New two-dimensional zinc oxide nanosheets: Properties, stability, and interconversion. Mater. Lett. 2020, 275, 128067 10.1016/j.matlet.2020.128067.

[ref39] Abdullahi Y. Z.; Ersan F. Stability and electronic properties of XO (X = Be, Mg, Zn, Cd) biphenylene and graphenylene networks: A first-principles study. Appl. Phys. Lett. 2023, 123, 252104 10.1063/5.0176681.

[ref40] Abdullahi Y. Z.; Tigli A.; Ersan F. Dodecagonal Zinc Oxide (d - Zn O) Monolayer for Water Desalination and Detection of Toxic Gases. Phys. Rev. Appl. 2023, 19, 014019 10.1103/PhysRevApplied.19.014019.

[ref41] Martins N. F.; Fabris G. S. L.; Maia A. S.; Albuquerque A. R.; Sambrano J. R. Inorganic graphenylene-like silicon carbide as anode material for Na batteries. FlatChem 2022, 35, 100410 10.1016/j.flatc.2022.100410.

[ref42] Martins N. F.; Maia A. S.; Laranjeira J. A. S.; Fabris G. S. L.; Albuquerque A. R.; Sambrano J. R. Hydrogen storage on the lithium and sodium-decorated inorganic graphenylene. Int. J. Hydrogen Energy 2024, 51, 98–107. 10.1016/j.ijhydene.2023.10.328.

[ref43] Lopes Lima K. A.; Ribeiro L. A. A DFT study on the mechanical, electronic, thermodynamic, and optical properties of GaN and AlN counterparts of biphenylene network. Mater. Today Commun. 2023, 37, 107183 10.1016/j.mtcomm.2023.107183.

[ref44] Monteiro F. F.; Giozza W. F.; de Sousa Júnior R. T.; de Oliveira Neto P. H.; Júnior L. A. R.; Júnior M. L. P. On the mechanical, electronic, and optical properties of the boron nitride analog for the recently synthesized biphenylene network: a DFT study. J. Mol. Model. 2023, 29, 215 10.1007/S00894-023-05606-4. 37347316

[ref45] Laranjeira J. A. S.; Abdullahi Y. Z.; Ersan F.; Sambrano J. R. ZnS and CdS counterparts of biphenylene lattice: A density functional theory prediction. Comput. Theor. Chem. 2024, 1235, 114580 10.1016/j.comptc.2024.114580.

[ref46] Prete M. S.; Pulci O.; Bechstedt F. Strong in- and out-of-plane excitons in two-dimensional InN nanosheets. Phys. Rev. B 2018, 98, 235431 10.1103/PhysRevB.98.235431.

[ref47] Liang D.; Quhe R.; Chen Y.; Wu L.; Wang Q.; Guan P.; et al. Electronic and excitonic properties of two-dimensional and bulk InN crystals. RSC Adv. 2017, 7, 42455–42461. 10.1039/C7RA07640A.

[ref48] Sun X.; Yang Q.; Meng R.; Tan C.; Liang Q.; Jiang J.; et al. Adsorption of gas molecules on graphene-like InN monolayer: A first-principle study. Appl. Surf. Sci. 2017, 404, 291–299. 10.1016/j.apsusc.2017.01.264.

[ref49] Yeganeh M.; Kafi F.; Boochani A. Thermoelectric properties of InN graphene-like nanosheet: A first principle study. Superlattices Microstruct. 2020, 138, 106367 10.1016/j.spmi.2019.106367.

[ref50] Dovesi R.; Erba A.; Orlando R.; Zicovich-Wilson C. M.; Civalleri B.; Maschio L.; et al. Quantum-mechanical condensed matter simulations with CRYSTAL. Wiley Interdiscip. Rev.: Comput. Mol. Sci. 2018, 8, e1360 10.1002/wcms.1360.

[ref51] Perdew J. P.; Burke K.; Ernzerhof M. Generalized Gradient Approximation Made Simple. Phys. Rev. Lett. 1996, 77, 3865 10.1103/PhysRevLett.77.3865. 10062328

[ref52] Krukau A. V.; Vydrov O. A.; Izmaylov A. F.; Scuseria G. E. Influence of the exchange screening parameter on the performance of screened hybrid functionals. J. Chem. Phys. 2006, 125, 224106 10.1063/1.2404663. 17176133

[ref53] Rothballer J.; Bachhuber F.; Rommel S. M.; Söhnel T.; Weihrich R. Origin and effect of In–Sn ordering in InSnCo 3 S 2: a neutron diffraction and DFT study. RSC Adv. 2014, 4, 42183–42189. 10.1039/C4RA03800B.

[ref54] Dovesi R.; Causa’ M.; Orlando R.; Roetti C.; Saunders V. R.; Causa’ M.; et al. Ab initio approach to molecular crystals: A periodic Hartree–Fock study of crystalline urea. J. Chem. Phys. 1990, 92, 7402–7411. 10.1063/1.458592.

[ref55] Ferrero M.; Ŕrat M.; Kirtman B.; Dovesi R. Calculation of first and second static hyperpolarizabilities of one- to three-dimensional periodic compounds. Implementation in the CRYSTAL code. J. Chem. Phys. 2008, 129, 244110 10.1063/1.3043366. 19123498

[ref56] Bader R. F. W.; Nguyen-Dang T. T. Quantum Theory of Atoms in Molecules–Dalton Revisited. Adv. Quantum Chem. 1981, 14, 63–124. 10.1016/S0065-3276(08)60326-3.

[ref57] Gatti C. Chemical bonding in crystals: New directions. Z. Kristallogr. 2005, 220, 399–457. 10.1524/zkri.220.5.399.65073.

[ref58] Wang V.; Tang G.; Liu Y. C.; Wang R. T.; Mizuseki H.; Kawazoe Y.; et al. High-Throughput Computational Screening of Two-Dimensional Semiconductors. J. Phys. Chem. Lett. 2022, 13, 11581–11594. 10.1021/acs.jpclett.2c02972. 36480578

[ref59] Grimme S.; Bannwarth C.; Shushkov P. A Robust and Accurate Tight-Binding Quantum Chemical Method for Structures, Vibrational Frequencies, and Noncovalent Interactions of Large Molecular Systems Parametrized for All spd-Block Elements (Z = 1–86). J. Chem. Theory Comput. 2017, 13, 1989–2009. 10.1021/acs.jctc.7b00118. 28418654

[ref60] Hourahine B.; Aradi B.; Blum V.; Bonafé F.; Buccheri A.; Camacho C.; et al. DFTB+, a software package for efficient approximate density functional theory based atomistic simulations. J. Chem. Phys. 2020, 152, 124101 10.1063/1.5143190. 32241125

[ref61] Bannwarth C.; Caldeweyher E.; Ehlert S.; Hansen A.; Pracht P.; Seibert J.; et al. Extended tight-binding quantum chemistry methods. Wiley Interdiscip. Rev.: Comput. Mol. Sci. 2021, 11, e1493 10.1002/wcms.1493.

[ref62] Berendsen H. J. C.; Postma J. P. M.; Van Gunsteren W. F.; Dinola A.; Haak J. R. Molecular dynamics with coupling to an external bath. J. Chem. Phys. 1984, 81, 3684–3690. 10.1063/1.448118.

[ref63] Born M.; Huang K.; Lax M. Dynamical Theory of Crystal Lattices. Am. J. Phys. 1955, 23, 474 10.1119/1.1934059.

[ref64] Peng Q.; Sun X.; Wang H.; Yang Y.; Wen X.; Huang C.; et al. Theoretical prediction of a graphene-like structure of indium nitride: A promising excellent material for optoelectronics. Appl. Mater. Today 2017, 7, 169–178. 10.1016/j.apmt.2017.03.001.

[ref65] Li C.; Fan B.; Li W.; Wen L.; Liu Y.; Wang T.; et al. Bandgap engineering of monolayer MoS2 under strain: A DFT study. J. Korean Phys. Soc. 2015, 66, 1789–1793. 10.3938/jkps.66.1789.

[ref66] Mortazavi B.; Rabczuk T. Anisotropic mechanical properties and strain tuneable band-gap in single-layer SiP, SiAs, GeP and GeAs. Phys. E 2018, 103, 273–278. 10.1016/j.physe.2018.06.011.

[ref67] Li F.; Tu K.; Zhang H.; Chen Z. Flexible structural and electronic properties of a pentagonal B2C monolayer via external strain: a computational investigation. Phys. Chem. Chem. Phys. 2015, 17, 24151–24156. 10.1039/C5CP03885E. 26315808

[ref68] Ferrero M.; Rérat M.; Orlando R.; Dovesi R.; Bush I. J. Coupled perturbed Kohn-Sham calculation of static polarizabilities of periodic compounds. J. Phys.: Conf. Ser. 2008, 117, 012016 10.1088/1742-6596/117/1/012016.

